# Partnership capacity for community health improvement plan implementation: findings from a social network analysis

**DOI:** 10.1186/s12889-016-3194-7

**Published:** 2016-07-13

**Authors:** J. Mac McCullough, Eileen Eisen-Cohen, S. Bianca Salas

**Affiliations:** School for the Science of Health Care Delivery, Arizona State University, 550 N 3rd Street, Phoenix, AZ 85004 USA; Maricopa County Department of Public Health, 4041 N Central Avenue, Phoenix, AZ 85012 USA

**Keywords:** Community health improvement plan, Social network analysis, Local health department

## Abstract

**Background:**

Many health departments collaborate with community organizations on community health improvement processes. While a number of resources exist to plan and implement a community health improvement plan (CHIP), little empirical evidence exists on how to leverage and expand partnerships when implementing a CHIP. The purpose of this study was to identify characteristics of the network involved in implementing the CHIP in one large community. The aims of this analysis are to: 1) identify essential network partners (and thereby highlight potential network gaps), 2) gauge current levels of partner involvement, 3) understand and effectively leverage network resources, and 4) enable a data-driven approach for future collaborative network improvements.

**Methods:**

We collected primary data via survey from *n* = 41 organizations involved in the Health Improvement Partnership of Maricopa County (HIPMC), in Arizona. Using the previously validated Program to Analyze, Record, and Track Networks to Enhance Relationships (PARTNER) tool, organizations provided information on existing ties with other coalition members, including frequency and depth of partnership and eight categories of perceived value/trust of each current partner organization.

**Results:**

The coalition’s overall network had a density score of 30 %, degree centralization score of 73 %, and trust score of 81 %. Network maps are presented to identify existing relationships between HIPMC members according to partnership frequency and intensity, duration of involvement in the coalition, and self-reported contributions to the coalition. Overall, number of ties and other partnership measures were positively correlated with an organization’s perceived value and trustworthiness as rated by other coalition members.

**Conclusions:**

Our study presents a novel use of social network analysis methods to evaluate the coalition of organizations involved in implementing a CHIP in an urban community. The large coalition had relatively low network density but high degree centralization—meaning key organizations link organizations otherwise not tightly partnering. Coalition members rated each other highly on trust, a positive sign for future partnership development efforts. Examination of network maps reveal key organizations that can be targeted for future partnership facilitation and expansion. Future network data collection will enable exploration of longitudinal trends and exploration of network characteristics versus health behavior, status, and outcome changes.

## Background

One of the core functions of public health is assessing the health of a community [1]. Often this is done by health departments in collaboration with partners from the public health system [2, 3]. Health departments seeking accreditation must complete a community health assessment (CHA) and community health improvement plan (CHIP)—collectively referred to as community health improvement processes—at least every 5 years [4]. More than two-thirds of local health departments (LHDs) have completed a CHA within the past 5 years and 56 % of LHDs have completed a CHIP [5]. Data from Florida suggest that while CHAs were a fairly ubiquitous activity for LHDs (due to statutory requirements), producing a written CHIP was far less common (21 % of LHDs as of 2007), with the majority of LHDs reporting low or moderate capacity for implementing strategies from a CHA [6]. Similar patterns were observed in Kansas [7].

CHAs and CHIPs can promote a virtuous cycle of identification, analysis, and prioritization of community needs, leading to implementation of shared goals for health improvement within a community [8]. Yet almost invariably, CHIPs represent a partnership between multiple organizations, meaning that health departments must be prepared to effectively engage a robust network of community partner organizations [9]. Partnering to complete a CHA or CHIP is a relatively common way in which LHDs work with community organizations [10]. Those partnerships are perceived as very important to overall CHA or CHIP activities [7]. In general, partnerships to complete a CHA or implement a CHIP involve a wider array of partners than other forms of LHD partnerships [9].

Evidence from Washington [11] and Wisconsin [9] shows that engaging community partners can be key to the success of health assessment and planning processes. Additionally, conducting a CHA can lead to new or strengthened relationships between a health department and partner organizations [11].

While a number of resources exist for LHDs to plan and implement a CHIP [12–14] little empirical evidence exists on how to leverage and expand partnerships to successfully implement a CHIP. A challenge commonly noted regarding community health improvement processes is that the typical program evaluation metrics—including changes in health behaviors, status, or outcomes—are not entirely applicable [15]. At its core, a CHIP centers on collaboration between health departments and community partners to coordinate and target resources effectively [16]. Often the ultimate goal of these collaborative efforts is improved population health, but data are scarce on the causal nature of the relationship between partnerships and improved health behaviors, status, or outcomes [17]. More information is needed about the black box that connects a CHIP to a change in health behaviors, status, or outcomes.

As opposed to CHAs, which are often LHD-led, community partners increasingly take on lead roles in CHIPs [6]. The role of the LHD may therefore shift to that of a trusted convener of a community network. Research in multiple settings has found that community health improvement processes (e.g., CHAs and CHIPs) are viewed more favorably by those that are involved in the processes than those that are not involved [15, 18]. Yet fostering effective public-private partnerships across multiple sectors can be difficult and is not always sustainable [19]. Research on the effectiveness of public health system networks has found that bigger is not always better. As networks get overly large, their effectiveness plateaus and eventually declines [20]. Therefore, more evidence on how to balance network size, density, and partners is needed. Currently no empirical data exist regarding the ideal size of a CHIP network, but these findings should motivate LHDs to thoughtfully assess and manage a CHIP network in order to effectively serve in their roles as trusted conveners. This makes understanding the network of partners engaged in a CHIP process all the more important. Indeed, much of the existing literature on the impact and effectiveness of community partnerships focuses on end-points such as health behaviors, status, or outcomes [17]. Yet given their frequent role as conveners of these networks [6] public health practitioners should also pay particular attention to characteristics of the network itself.

One established method for measuring and analyzing network characteristics is social network analysis [21]. Network analyses have been used to explore public health topics ranging from patterns of disease transmission [22, 23] to emergency preparedness [24] to administrative structures within public health departments [25]. To date, network analysis methods have not been used to specifically explore the structural and relational aspects of the partners working to implement a CHIP.

This study synthesizes an ongoing multi-year, multi-part evaluation of the implementation of a CHIP through a community-wide partnership known as the Health Improvement Partnership of Maricopa County (HIPMC) in Arizona, a coalition of approximately 75 organizations collaborating to address five priority areas from the Maricopa County Department of Public Health (MCDPH) CHA—Obesity, Diabetes, Cardiovascular Disease, Lung Cancer, and Access to Care—through public-private partnerships.

Maricopa County, AZ presents a unique opportunity to capture all community health improvement processes across a large, urban environment. Maricopa County comprises over 90 % of the Phoenix-Mesa-Glendale metropolitan statistical area (MSA), the 12th largest MSA in the U.S. The MCDPH is the only public health department in the county and has been actively involved in leading both the county’s CHA and CHIP processes. MCDPH provides 2.5 full time staff to coordinate the network, provide communication, and evaluation support.

The purpose of this paper is to identify characteristics of the network involved in implementing the CHIP in one large community at the onset of a CHIP intervention effort. This study explores the organizational relationships and networks present in the implementation of a CHIP by a large, urban LHD through the use of social network analysis. The main goals of the analysis are to: 1) identify essential network partners (and thereby highlight potential network gaps), 2) gauge current levels of partner involvement, 3) understand and effectively leverage network resources, and 4) enable a data-driven approach for future HIPMC network improvements. This network will be tracked over time, including monitoring membership, engagement, relationships, and ultimately changes in health behaviors, status, and outcomes.

## Methods

This study gathered primary data collected from surveys of HIPMC partner organizations.

### HIPMC Background

**Fig. 1:**
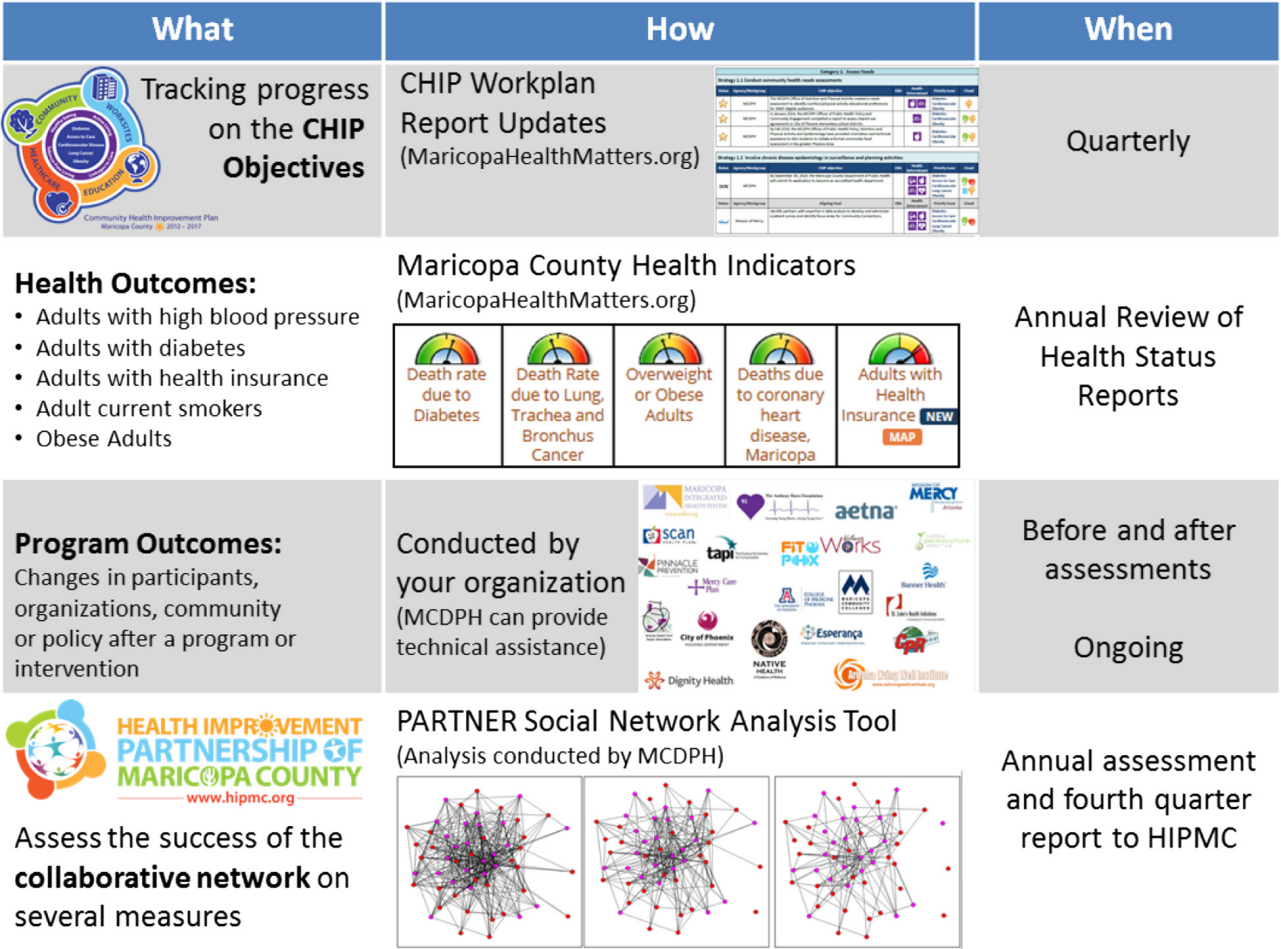
Evaluation schematic for Maricopa County Department of Public Health community health improvement plan

The HIPMC is the network of organizations partnering to implement the CHIP in Maricopa County (greater Phoenix, Arizona metro area). Maricopa County has a population of approximately 4 million persons and is served at the local level by the MCDPH. The county’s CHA was completed in June 2012, after an 18-month engagement process involving a range of community stakeholders and partners. The CHA enabled development and deployment of the county’s CHIP in February 2013. HIPMC grew out of the community-centered CHA and CHIP processes and formally convened in late 2013. The HIPMC convenes approximately quarterly as one large group; each meeting includes multiple discussion topics. Many LHDs have likely established similar CHIP strategies and evaluation plans. As shown in Fig. 1, MCDPH has constructed a wide-ranging evaluation framework to assess process and outcome measures, and health condition surveillance.

A major distinguishing element of the HIPMC’s evaluation plan is the purposeful measurement of the network of partners contributing to the effort. In addition to monitoring the health outcomes and program outcomes attributable to the HIPMC, the success of the collaborative network was also assessed using several measures. To gather data on existing network connections, a survey was distributed in early 2014 to all HIPMC members. Regular follow-up surveys are planned for the duration of the CHIP period (through 2017).

### Survey instrument & design

Data were obtained through an online survey of HIPMC members using the Program to Analyze, Record, and Track Networks to Enhance Relationships (PARTNER) Tool [26]. This tool measures key aspects of partnerships and connectivity and has been previously used in many public health collaboratives [27, 28]. Each organization was surveyed along eight dimensions regarding their organization’s relationships (if any) with every other HIPMC member organization: (1) frequency of interaction, (2) level of collaboration, (3) perceived power and influence, (4) perceived level of involvement, (5) perceived resource contributions, (6) perceived reliability, (7) extent of shared vision, and (8) openness to discussion. In addition to these survey items, other basic information was obtained. A copy of the survey instrument used is available in the online appendix.

Surveys were distributed via e-mail to chief HIPMC points-of-contact at each member organization (*n* = 53). Respondents were given 2 weeks to reply and were sent a reminder email. For those organizations that still hadn’t responded, phone calls from MCDPH staff were made to organizational contacts requesting their immediate participation.

### Analysis

Data analysis—including social network mapping—was performed using the PARTNER Tool [26]. The main quantitative outcomes of interest for the overall network were: Density (percentage of ties present in the network in relation to the total number of possible ties in the entire network), Degree Centralization (the lower the centralization score, the more similar the members are in terms of their number of connections to others), and Trust (percentage of how much members trust one another. A 100 % occurs when all members trust others at the highest level). These three numeric scores also enable comparison of HIPMC network characteristics over time.

Scores were also calculated for all individual HIPMC coalition members. Dimensions surveyed included Degree Centrality (the extent to which the network is centered around only a few members versus having many members at the center of the network with equal number of relationships), Trust (the extent to which an organization was judged by other HIPMC members as being reliable in following through on commitments, as having a shared vision with the HIPMC, and as being open to discussion), and Power (the extent to which an organization holds a prominent position in the community being powerful, having influence, success as a change agent, and showing leadership). Network maps were also created for these select areas, again using the PARTNER tool. This research study was determined by the Arizona State University Institutional Review Board to be exempt under 45 CFR 46.101(b)(5). The study complies with the Principles of the Ethical Practice of Public Health code.

## Results

A total of 41 HIPMC member organizations responded to the survey (response rate: 77 %). The overall HIPMC social network had a density score of 30 %, degree centralization score of 73 %, and trust score of 81 %.

**Fig. 2:**
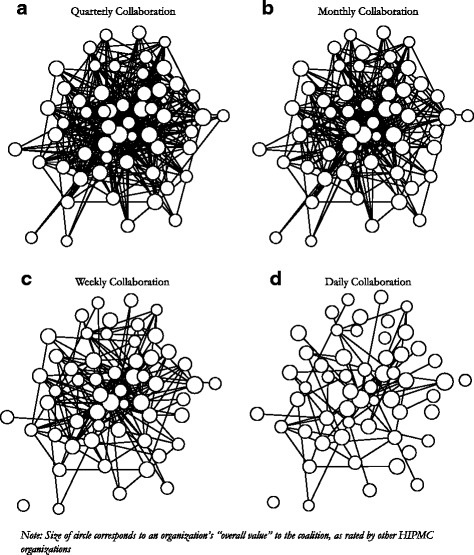
Collaboration among HIPMC member organizations occuring Quarterly (**a**), Monthly (**b**), Weekly (**c**), and Daily (**d**)

Network maps identified existing relationships between HIPMC members. Figure 2 shows the network of relationships between HIPMC member organizations according to how frequently the collaboration occurs (quarterly, monthly, weekly, and daily). The size of node corresponds to an organization’s overall value to the coalition, as rated by other HIPMC organizations, with larger nodes representing organizations rated as having higher value. Fewer network connections were observed for more frequent levels of collaboration (weekly or daily) versus less frequent levels of collaboration (monthly or quarterly). Visual inspection of the four network maps reveals organizations that are both perceived as valuable and involved in frequent collaboration with multiple HIPMC partners, and organizations that are perceived as valuable but not (yet) involved in frequent collaboration.

**Fig. 3:**
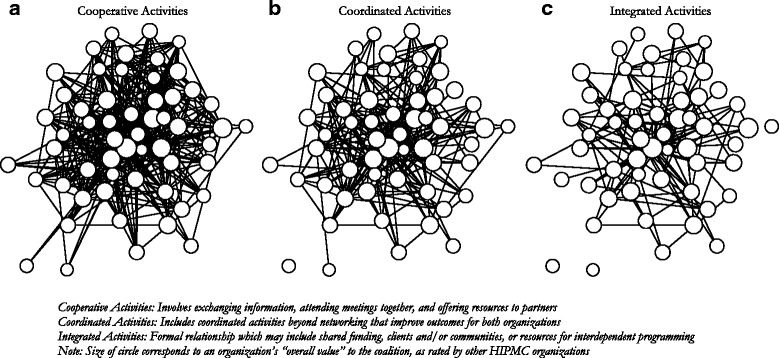
Collaborative relationships among HIPMC member organizations for Cooperative Activities (**a**), Coordinated Activities (**b**), and Integrated Activities (**c**)

Figure 3 shows the network of relationships between HIPMC member organizations according to the level of involvement of the relationship (cooperative, coordinated, and integrated). The size of the node corresponds to an organization’s overall value to the coalition, as rated by other HIPMC organizations. Fewer network connections were observed for more intensive levels of collaboration (integrated activities) versus less intensive levels (cooperative activities). As in Fig. 2, these network maps can also be used to identify organizations who are involved in more- or less-intensive collaborations with other HIPMC member organizations.

**Fig. 4:**
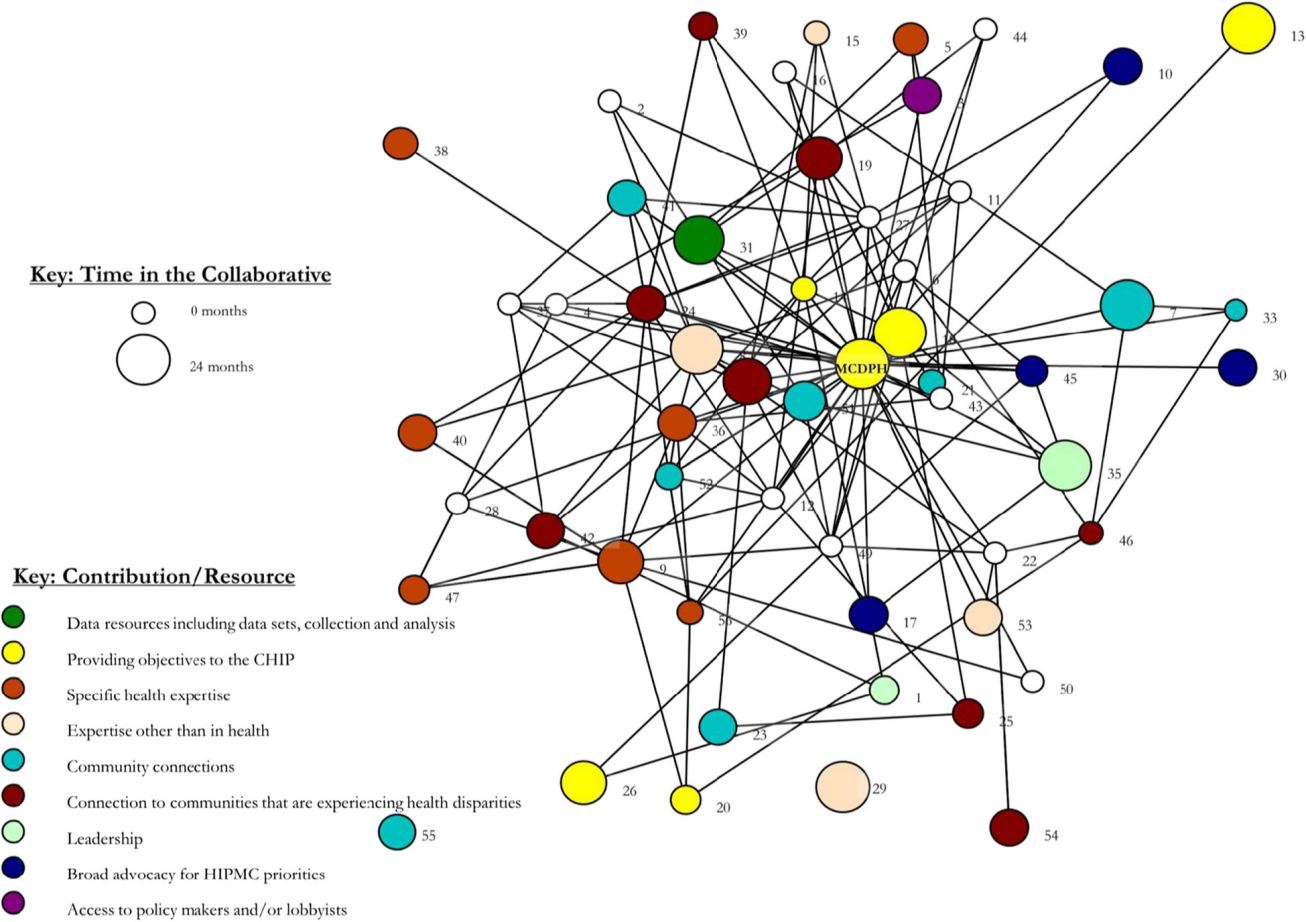
HIPMC coalition network map for integrated activities, by organization’s key contribution/resource and time participating in MCDPH-led community health improvement processes

Figure 4 shows the HIPMC’s network map for integrated activities by what each organization reported as their key contribution to the coalition. The size of the nodes in Fig. 4 corresponds to an organization’s length of time participating in MCDPH-led community health improvement processes (including county-wide CHA, CHIP, and/or HIPMC). Duration ranged from 0 (new members) to 24 months. Duration of an organization’s participation in HIPMC was associated with higher closeness centrality (r = 0.37, *p* = 0.01) and with higher overall value (r = 0.36, *p* = 0 .02) but was not significantly associated with total trust score.

Nine different key organizational contributions were reported by HIPMC members (out of 12 possible categories on the survey; the three contributions not self-reported by HIPMC members as their most important contribution to HIPMC were: *funding*, *in-kind resources* (e.g., meeting space), and *facilitation*). Visual inspection of the network map reveals existing partnerships and, importantly, relationships that do not currently exist. For example, none of the organizations engaged in *Broad advocacy for HIPMC priorities* have existing integrated relationships with the organization that offers *Access to policy makers and/or lobbyists*. Likewise, three of the four organizations that contribute *Expertise other than in health* do not currently have integrated activities underway with organizations that contribute *Specific health expertise*. These exemplar pairs of HIPMC contributions are two of many such comparisons that can be made and are highlighted here for illustrative purposes of where HIPMC partnership building efforts may be targeted.

Characteristics of individual HIPMC organizations, as rated by other HIPMC members, are shown in Table 1. With the exception of MCDPH, organizational names have been redacted. Organizational numbers in Table 1 correspond to those pictured in Fig. 4. Organizations are listed in descending order of number of ties reported with other HIPMC members. The final eight columns in Table 1 show average scores (1 – 4) for each organization along eight important dimensions related to partnership and network capacity. Highlighted cells correspond to organizations scoring in the top quartile for each category. While many organizations with high levels of network connectivity scored highly in at least one of the eight perceived value categories, several organizations with lower network connectivity also received strong scores. For example, organization 27 ranked 10th from the bottom in number of connections, but scored in the top quartile for 7 of the 8 perceived value categories. Organization 29 scored in the top quartile in all 8 categories and has multiple years of participation in community health improvement processes, yet reported no integrated activities with other coalition members.

**Table 1:**
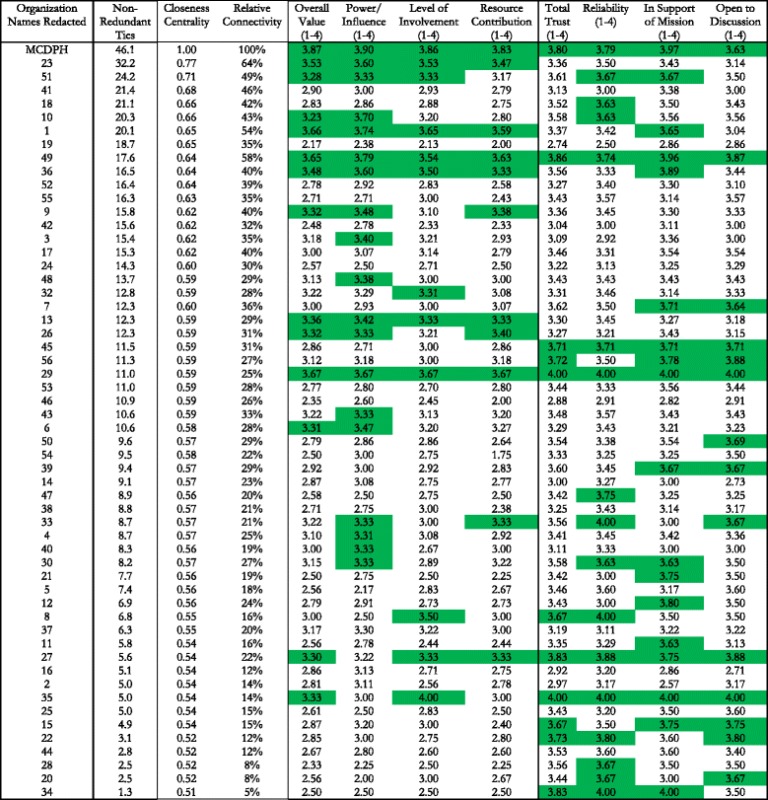
Centrality, connectivity, value, and trust scores for individual organizations in the HIPMC coalition

Note: Highlighted cells represent scores in the top quartile for each column measure

Definitions of Terms Used in Table

*Degree Centrality:* number of connections to other members of the network

*Non-redundant ties:* number of non-redundant ties in relation to the other members that each organization is connected to

*Closeness Centrality:* Measures how far each member is from other members of the network in terms of # of links between each member. A high score (close to 1) indicates members who have the shortest ‘distance’ between all other members

*Relative Connectivity:* Based on measures of value, trust, and # of connections to others, the connectivity score indicates the level of benefit an organization receives as a network member, in relation to the member with the highest level of benefit (100 %)

*Overall Value:* Average of the ranking given by all other members for that organization along three dimensions: authority, influence, and impact. (Scale of 1–4)

*Total Trust:* Average of the ranking given by all other members for that organization along three dimensions: reliability, support of mission, and open to discussion. (Scale of 1–4)

Table 2 shows the correlation coefficients for three measures of an organization’s existing relationships with other HIPMC organizations (number of non-redundant ties, closeness centrality, and relative connectivity) and eight measures of that organization’s value, contributions, and trustworthiness. Overall value and power/influence were both positively correlated with all three measures while relative connectivity was positively correlated with an organization’s level of involvement in the coalition and value of its resource contributions.

**Table 2:** Correlation coefficients between organization’s HIPMC relational measures and perceived value to the coalition

	Number of ties	Closeness centrality	Relative connectivity
Overall Value	0.48^a^	0.48^a^	0.58^a^
Power/Influence	0.49^a^	0.47^a^	0.58^a^
Level of Involvement	0.42	0.42	0.49^a^
Resource Contribution	0.40	0.40	0.50^a^
Total Trust	−0.02	0.03	0.07
Reliability	−0.08	−0.03	−0.03
Support of Mission	0.14	0.17	0.24
Open to Discussion	−0.13	−0.08	−0.04

^a^Signifies correlation is significant at *p* < .05 level, after Bonferroni correction

Finally, Table 3 shows network metrics of interest according to organization’s self-reported most important contribution to the HIPMC. Due in part to small sample sizes, no significant differences were observed across the nine response categories in terms of mean closeness centrality, overall value, or total trust scores.

**Table 3:** Network scores on closeness centrality, overall value, and total trust, according to organization’s most significant contribution to the HIPMC coalition network

Organization’s most important contribution to HIPMC (Self-Rated)	Number of respondents	Organizations’ mean scores for:		
		Closeness centrality	Overall value	Total trust
Broad advocacy for HIPMC priorities	4	0.57	2.98	3.53
Community connections	11	0.60	3.01	3.49
Connection to communities that are experiencing health disparities	8	0.60	2.69	3.24
Data resources including data sets, collection and analysis	1	1.00	3.87	3.80
Expertise other than in health	3	0.59	2.81	3.46
Leadership	7	0.58	3.08	3.39
Providing objectives to the CHIP	4	0.57	3.03	3.25
Specific health expertise	7	0.58	3.02	3.50
Unknown (not reported or missing)	11	0.58	2.96	3.47
TOTAL	56	0.60	2.97	3.43

Note: Differences between groups were not significant at p = .05 level

## Discussion

Community health improvement processes are important and widely used approaches for assessing and improving the health of communities. Through the dedicated work of health department practitioners and community partner organizations, meaningful gains can be made in health behaviors, status, and outcomes [1–3]. Yet there is currently little empirical evidence evaluating the CHIP implementation structure. This study represents a first-look at one large urban community’s evaluation of the network underlying its CHIP implementation effort.

Overall, the HIPMC network had high levels of trust and centralization and low density. In practice, this represents both a challenge and an opportunity. Having a high degree of centralization means that a relatively small number of organizations link other coalition members who would otherwise not be connected [29]. Communication or collaboration within the network may thus rely on these organizations, making these organizations key partners within the HIPMC. These organizations can be gatekeepers of partnerships and coordinated activities with other HIPMC members. Through targeted engagement, MCDPH can help to ensure that these key gatekeepers remain productively engaged with the HIPMC and continue to be able to link other organizations that may otherwise be less connected to the coalition. This represents a potentially efficient way to disseminate information throughout the network, but also may present bottlenecks if the information *has* to flow through multiple organizations before reaching all coalition members.

Examination of position within the network and number of organizational ties also identified several individual organizations that, based on high value and trust scores, may be important viable candidates for further engagement (e.g., organization 27 from Table 1). Likewise, Table 2’s findings show a strong positive relationship between network connectivity measures and an organization’s value to the coalition. A similar relationship between connectivity measures and total trust was not observed. While correlation does not imply causation, future work to grow or strengthen the network may benefit from focusing on organizations that are highly valued rather than those that are trusted but not highly valued.

The HIPMC’s network density also presents both a challenge and an opportunity. Compared to other networks, our network has relatively low density (percent of all possible links between network members) [29]. Given the MCDPH’s role as convener of the HIPMC coalition (and the most connected to other HIPMC members, according to our findings), dedicated efforts to establish new linkages may be beneficial. Yet these efforts must be purposeful and circumspect, as research shows that more connections within a network are better only to a point. When the network gets too large and interconnected, additional connections can provide little or no additional benefits [20].

The high level of trust within the HIPMC coalition represents a critical strategic asset for network success. Trust can be challenging to build within a network. In its absence, efforts to optimize density and centralization may face meaningful barriers. Sustaining high levels of trust should become a key priority for coalition leaders moving forward. The network of organizations engaged for a CHIP by a LHD represents an asset that may promote CHIP success if successfully managed or hinder CHIP success if not successfully managed.

The size of the HIPMC network (*n* = 53) was far larger than has been reported in other settings. The mean number of partnerships from a statewide survey in Wisconsin was eight [9] while a broad partnership-focused initiative in California included two to five partners per health department [3]. This difference is likely at least partially due to the fact that Maricopa County represents the third largest local health jurisdiction in the country—over 4 million individuals. It may also be due to the fact that the network is focused on multiple specific priority areas identified through the CHA, making the network of relevant community stakeholders broad and diverse. This underscores the importance of understanding the characteristics of the HIPMC social network and strategically managing partner connections. One potential challenge this may present is that empirical research has demonstrated decreasing returns to scale with network size—meaning bigger is not always better [20]. Particular attention is needed to ensure efficient coordination and information sharing among the broad array of HIPMC partners.

We found no evidence that an organization’s most important contribution to the HIPMC (as self-reported by each respondent) was associated with its position in the HIPMC social network, its overall value, or its trustworthiness. While this may be due at least in part to small sample sizes for each of the various contribution types, it is instructive for this network to note that organizations were centrally located, highly valued, and highly trusted across contribution types. We interpreted this to imply that organizational characteristics besides its contribution type played an important role in determining its position and value for the network. In contrast to the finding that contribution type did not seem to determine network position or value, length of participation in the HIPMC was significantly associated with higher centrality (more connections) and higher overall value. The causal nature of this association is unknown as it is plausible that more connected and valued organizations joined the HIPMC earlier than less connected or valued organizations. Regardless, this finding can inform stakeholder retention activities by stressing the importance of organizations that have been long-standing members of the coalition.

Using the network maps and organizational scores obtained from this data collection, we were able to identify organizations that can potentially be targeted to sustain and strengthen the HIPMC coalition. In our network, organizations with more network connectivity ranked highly in overall value and power/influence—and according to one connectivity measure, also ranked highly in level of involvement and resource contribution. Yet one organization (29) scored in the top quartile of all eight organizational value categories and reported being a relatively long-serving coalition member, but reported no integrated partnership activities with other HIPMC members. Another organization (27) scored in the top quartile of seven of eight value and trust measures yet had few reported organizational connections within HIPMC. These and other anecdotal findings can help dedicated HIPMC to expand the quantity and/or quality of network ties and facilitate a robust set of partnerships by targeting trusted and valued organizations. Given that we found a positive correlation between organizational value and network connectivity, further work to unpack the direction of this association may be helpful (i.e., does value cause connectivity or vice versa). Another iteration of this survey is scheduled to be deployed to examine whether and how connectivity, value, and trust change among HIPMC members over time; it is MCDPH’s intention to repeat this network analysis process approximately every year.

Study findings can also be employed by MCDPH to examine our own position and scores within the network. Overall, MCDPH ranked at the top in terms of connectedness to other network members. As the convening organization, this is perhaps not surprising. MCDPH also ranked at the top in terms of overall value. We interpreted this as a positive sign regarding MCDPH’s work with HIPMC and the community. While MCDPH still ranked highly in trust measures (top quartile of organizations across all four measures), this may be an area for further exploration and effort on MCDPH’s part. For example, MCDPH ranked fifteenth in terms of openness to discussion. Not a bad showing, but given strong rankings in other categories, this would be an area for potential improvement. It should be noted that since MCDPH was the one administering the survey, social desirability response bias may be an issue [30].

Our findings should be viewed in light of the study’s limitations. First, this study represents the experiences of one CHIP implementation process, led by one LHD, in one community. Many setting-specific factors may limit generalizability of findings to other settings. We also relied on a widely used and readily available assessment instrument (PARTNER tool) for data collection. A major contribution of this paper is the presentation of social network analysis methods as a potentially valuable tool for assessing coalitions involved in community health improvement processes. Second, as a social network analysis, our study may be sensitive to missing data from non-respondents and other non-HIPMC participant organizations [31]. However, studies have shown network measures to be adequately robust to missing data [32]. Third, organizations were not surveyed on their network partnerships in the initial stages of the HIPMC, but after several years of community health improvement processes (CHA and CHIP) where partnerships could potentially be established and strengthened. Thus the network snapshot represents the collaborative resources available at a single and context-specific point in time. Fourth, this analysis was mainly quantitative in nature. Future exploration of community partnerships may benefit from use of mixed methods approaches to lend context to quantitative findings. Finally, as one component in a larger evaluation framework, the ultimate impacts of this coalition are unknown. While we are of course hopeful that this CHIP implementation effort will be fruitful, its potential impacts on health behaviors, status, and outcomes are currently unknown.

## Conclusion

This study presents an analytic framework and initial results for a crucial component of CHIP implementation—the strength of the collaborative network of partners. CHIPs are ultimately aimed at population health improvement, yet very little empirical data exist to link CHA or CHIP partnerships to improved population health outcomes. The ‘black box’ between presence of CHIP partnerships and improved outcomes is formidable. Better understanding the characteristics of the public health system network charged with implementing a CHIP will help shed light on the capabilities of the partnerships to positively impact population health in the communities served.

The HIPMC network is a large coalition that shares high levels of trust among its member organizations. Its relatively low density and high degree of centralization means that there are key organizations that link organizations that might not otherwise be collaborating.

Like many efforts in public health, HIPMC must target its limited resources effectively. This analysis enabled the identification of organizations strategically positioned within the current coalition network (e.g., organizations involved in integrated activities as shown in Fig. 3 and/or those with high closeness centrality scores as shown in Table 1) or those who are highly regarded but are only currently collaborating with a select few coalition members. Paying particular attention to these critical organizations, next steps involve working closely with coalition members to promote additional ties and more intensive ties.

By observing the maps of the types of activity relationships in the network, key community participants and MCDPH staff have determined the need for increased integrated activities to strengthen the HIPMC network, with the ultimate goal of influencing the health measures on the CHIP. This data-driven approach has led to the formation of a proposed mini-grant opportunity to support and encourage integrated activities for the network—a major departmental priority. Additionally, MCDPH staff can use the findings from this network analysis to purposefully facilitate the generation of new partnerships or the strengthening of existing partnerships between current HIPMC members using a data-driven approach (i.e., each organization’s responses to the survey).

Future waves of data collection and analysis are planned in order to observe changes in the partnership network and track these process outcomes across the implementation of the county’s CHIP. In addition, qualitative data will be collected to shed light on the characteristics and impacts of new and future HIPMC coalition partnerships.

## Abbreviations

CHA, community health assessment; CHIP, community health improvement plan; HIPMC, health improvement partnership of Maricopa County; MCDPH, Maricopa County Department of Public Health.
